# Within leaf variation is the largest source of variation in agroinfiltration of *Nicotiana benthamiana*

**DOI:** 10.1186/s13007-015-0091-5

**Published:** 2015-10-14

**Authors:** Hany Bashandy, Salla Jalkanen, Teemu H. Teeri

**Affiliations:** Department of Agricultural Sciences, Viikki Plant Science Centre, University of Helsinki, P.O. Box 27, 00014 Helsinki, Finland; Department of Genetics, Cairo University, 13 Gamaa St., Giza, 12619 Egypt

**Keywords:** Agrobacterium meditated transformation, Agroinfiltration, Luciferase expression, Nested design, Estradiol induction

## Abstract

**Background:**

Transient gene expression utilizing syringe agroinfiltration offers a simple and efficient technique for different transgenic applications. Leaves of *Nicotiana benthamiana* show reliable and high transformation efficiency, but in quantitative assays also a certain degree of variation. We used a nested design in our agroinfiltration experiments to dissect the sources of this variation.

**Results:**

An intron containing firefly luciferase gene was used as a reporter for agroinfiltration. A number of 6 week old tobacco plants were infiltrated for their top leaves, several samples were punched from the leaves after 2 days of transient expression, and protein extracts from the samples were repeatedly measured for luciferase activity. Interestingly, most of the variation was due to differences between the sampling spots in the leaves, the next important source being the different leaves on each plant. Variation between similar experiments, between plants and between repetitive measurements of the extracts could be easily minimized.

**Conclusions:**

Efforts and expenditure of agroinfiltration experiments can be optimized when sources of variation are known. In summary, infiltrate more plants but less leaves, sample more positions on the leaf but run only few technical replicates.

**Electronic supplementary material:**

The online version of this article (doi:10.1186/s13007-015-0091-5) contains supplementary material, which is available to authorized users.

## Background

A wide range of methods and techniques have been used to produce transient gene expression in plant cells for studying promoter activity, gene and protein function, or protein–protein interactions in vivo [1–4]. Protoplast transformation and particle bombardment date back furthest [5, 6] and in spite of their drawbacks in being time consuming and sometimes inefficient, they still are used because of their benefits [7]. For example, particle bombardment is targeted to intact tissues where different cell and tissue types can be distinguished for the assay. During more recent years, agrobacterium based transient assays have become more and more widely used [8–10]. Agrobacterium is the earliest [11, 12] and still today often the preferred gene transfer tool to generate stably transformed plants. Agrobacterium interacts with a wide range of plant cells and through a type IV secretion system injects a single stranded DNA molecule into the plant cell, which subsequently gets transported to the nucleus, made double stranded and finally gets integrated into a chromosomal position [13].

Interestingly, genes residing on the transferred DNA (T-DNA) are expressed early during the process and, according to the present view, prior to and independent of the integration event itself [14]. This early expression is transient and is strongly reduced after peaking at ca. 2 days [15]. Fading away of the transient expression is not due to fast degradation of non-integrated T-DNA, but an active silencing process. Coinfiltration of T-DNA from which viral silencing suppressor proteins are expressed prolongs transient expression by many days, highest accumulation levels occurring at around 6 or 7 days post infiltration [16, 17].

Agrobacterium based transient gene expression can take place in various tissues [9], but the most commonly used target is the mesophyll of expanded leaves. An agrobacterium suspension can be infiltrated with vacuum or a syringe to the parenchymal airspace, hence the method is referred to as “agroinfiltration”. Particularly leaves of *Nicotiana benthamiana* have proven to be rewarding targets for agroinfiltration. A large fraction of *N. benthamiana* mesophyll cells are transformed by agrobacterium and in the extreme cases as much as 50 % [18] of total soluble leaf protein can be encoded by the transferred gene. This has led to applications where pharmaceutically active proteins are produced by leaf infiltration at a commercially viable scale [19–21]. For research, proteins difficult to yield in microbial systems have been produced in *N. benthamiana* for their characterization [22–24] or allowing their function to take place in the plant cells leading to changes in metabolism clarifying their (enzymatic) roles or in formation of pharmaceutically or commercially interesting small molecules [25].

In addition to bulk protein production, syringe or vacuum agroinfiltration has been used to study protein–protein interactions and plant promoter function in vivo [1, 26]. For quantitative assays, variation originating from biological and technical sources limits the accuracy and statistical power of the assays. Compared to using stably transformed plant lines, transient expression assays already eliminate variation due to different chromosomal positions and epigenetic states of the transferred genes. Still, plenty of variation remains. In this work, we address the source of this variation by using a hierarchical (nested) experimental design, where components of the experimental variance can be teased apart. Our aim was to understand the source of the variation in order to design experiments that are optimal in respect to the effort and expense used. In short, our results show that most of the variation originates from within the infiltrated leaf (between sampling spots), position of the leaf on the plant being the second largest source.

## Results

### Experimental design

**Fig. 1:**
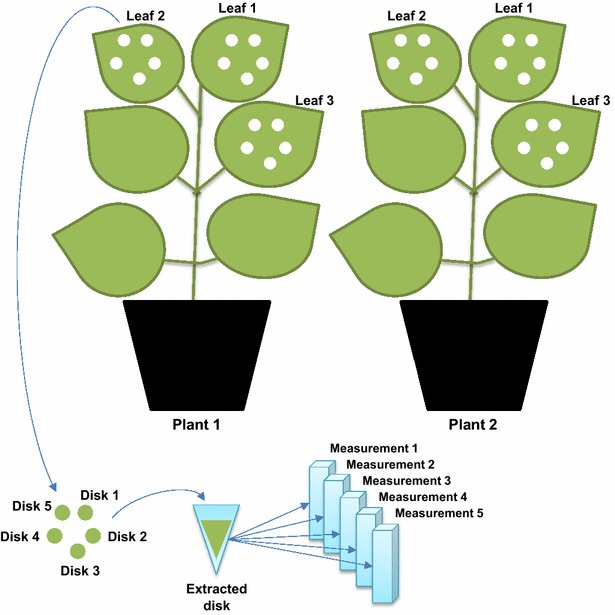
Design of the nested infiltration experiments. In the first experiment, three leaves of two *Nicotiana benthamiana* plants were infiltrated with agrobacterium, each leaf was sampled from five positions and luciferase activity of each sample extract was measured five times. The same procedure was repeated for four different promoters or inducer treatments driving the luciferase reporter

We ran two different experiments using a similar hierarchical design. Our original intention was to test estradiol induction of the XVE/LexA system [27] in agroinfiltrated *N. benthamiana*, compare the background and induced levels to the widely used Cauliflower Mosaic Virus 35S promoter [28], and to compare the G10-90 promoter [29], driving the XVE transcription factor, to the 35S promoter. Therefore, three constructs with the reporter gene encoding firefly luciferase (LUC) were used in this experiment. For each construct (for XVE-*LUC* with and without estradiol), two *N. benthamiana* plants were used, three top leaves were infiltrated from each plant, five samples were punched from each leaf and extracted, and each extract was measured five times for luciferase activity (technical replicates) (Fig. 1).

In the second experiment we used only a 35S-*LUC* construct. Three plants were treated, three top leaves were infiltrated, four samples were punched from each leaf and each sample measured twice. This was repeated three times with 1 week intervals (experimental replicates), giving the topmost hierarchical level of the second experiment.

All results were tabulated (Additional file 1: Tables S1, S2) and variance components were calculated as described in materials and methods.

### Promoter efficiencies in agroinfiltration

**Fig. 2:**
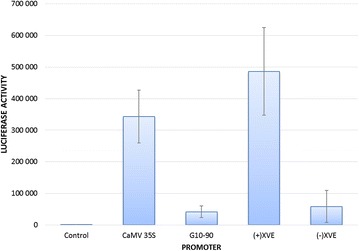
Promoter activity in agroinfiltrated *Nicotiana benthamiana* leaves. The estradiol inducible XVE/LexA cassette was measured with (+) and without (−) induction. *Error bars* show standard deviation of all measurements

Comparison of the three different promoters (XVE promoter for uninduced and induced levels) showed that, compared to the 35S promoter, XVE promoter gave an uninduced background level of 17 % and an induced level of 140 %. In this system, G10-90 promoter yielded luciferase activity that was 12 % of the 35S promoter driven activity (Fig. 2).

### Source of variation

**Fig. 3:**
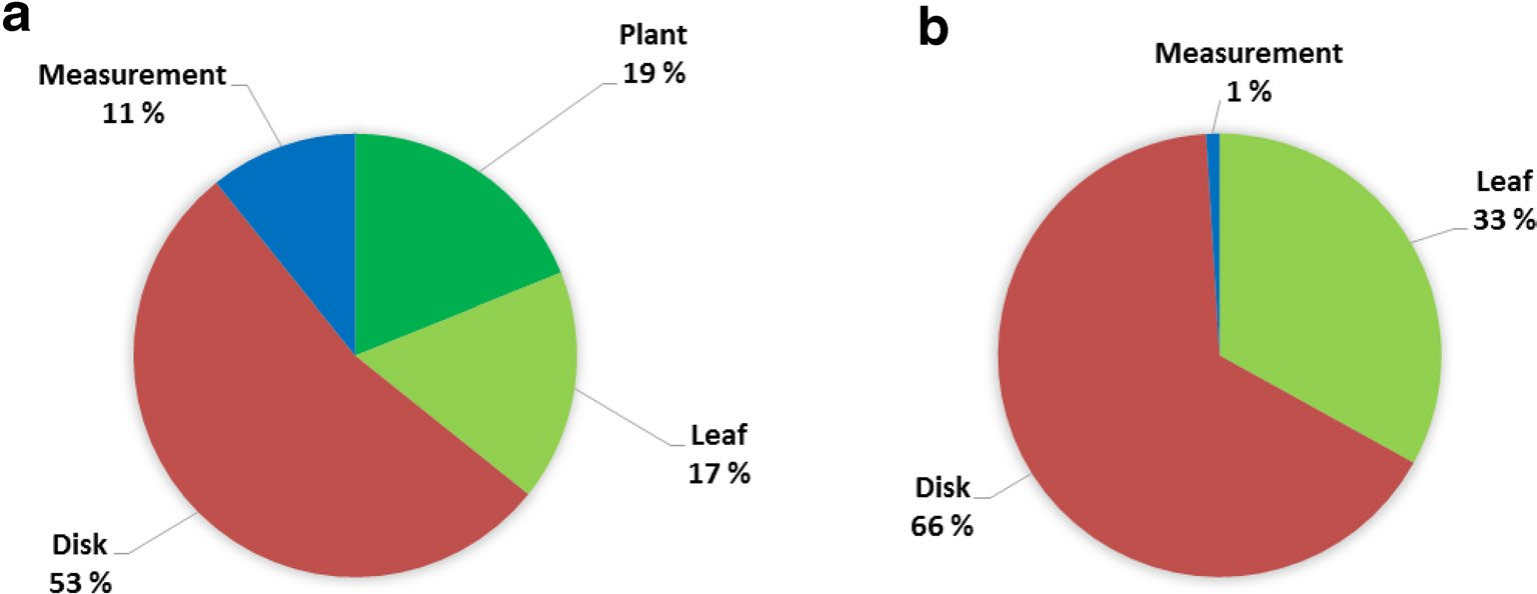
Components of variance in the agroinfiltration experiment. In the first experiment (**a**), the variance caused by the different promoters is excluded. In the second experiment (**b**) none of the observed variance could be addressed to the three plant individuals within one agroinfiltration subexperiment, or its three repetitions. In both experiments, largest variation occurred between the sample disks punched from infiltrated leaves

The hierarchical design of the promoter test experiment allowed us to split the total experimental variance to its components. Largest fraction of the variance (85 %) was due to the promoters (or induction conditions) applied, as expected. As the promoters cause a fixed effect, their contribution was ignored when inspecting the distribution of the remaining variance (Fig. 3a).The remaining variance concentrated to the within leaf sampling (between punch holes or disks, 53 %), to the leaf position (17 %) and to the plant individual (19 %). Inspecting results from individual plants used in the experiment showed that in few cases the two plants used for the experiment were not alike. Technical replication of the luciferase activity contributed least (11 %) to total variance.

The second experiment was designed to address the agroinfiltration variance in more detail by using a single reporter construct (35S-*LUC*) and more plants but less technical replicates. In the first experiment, only 0.5 µl of leaf extract was used for the luciferase assay. Although the variance of technical replication was smallest, part of it might be due to inaccurate pipetting. We increased the sample volume to 10 µl, but in order to keep the luciferase activity within the range of the luminometer, we mixed the reporter agrobacterium strain with one expressing the silencing suppressor p19 [15] in ratio 1:50. Silencing suppression is commonly used in agrobacterium infiltration and allows transient expression to continue for up to a week, however here the role of the second strain was simply to dilute the luciferase carrying agrobacterium. We also took extra care to choose plants identical in size and figure for the experiment, leaving the largest and smallest plants on the tray out of the experiment.

Analysing the second experiment for its variance components showed that increasing the volume pipetted for the luciferase assay nearly completely eliminated variance from technical replication (Fig. 3b). In addition, variance between the three plants in the experiment and between the three experimental replicates of the infiltration series was negligible. Similar to the first experiment, largest variation came from between samples punched from each leaf analysed (66 %) and next largest from leaves infiltrated within each plant (33 %).

To catch possible sources of the within leaf variation, we ran some additional controls. The agrobacterium suspension spreads seemingly evenly in the airspace of the expanded leaf but this does not assure that the bacteria are distributed evenly. To test this, infiltrated leaves were sampled as for the luciferase assay and bacteria were released by homogenisation. Plating of serial dilutions of the suspensions showed 12 % variation but no trend in respect to the distance from the infiltration spot (Additional file 2: Figure S1).

Buyel and Fischer [30] observed significant variation between sampling positions within agroinfiltrated *N. tabacum* leaves and their experiments showed a trend of increased transient expression towards the basal parts of the leaf. Two of the four sampling spots in our experiment were taken closer to the tip of the leaf and two closer to the base, but the variation observed could not be addressed to the sampling position (Additional file 2: Figure S2). Still, there was a slightly higher average level of expression closer to the tip of the leaf and variation within the tip samples was somewhat lower than between the basal samples (Additional file 2: Figure S2).

Finally, we tested if our protein extraction procedure causes variation. We repeatedly sampled test leaves and measured soluble protein content in the extracts. Variation was only 5.5 %, while for the transient luciferase expression it was 26 % within leaves, on average (Additional file 2: Figure S3). Although none of the tested sources contributed a major fraction of the within leaf variance, together they may contribute up to 15 % (Additional file 2: Figure S3).

The second largest source of variation comes from leaves within each infiltrated plant. In the second experiment we originally infiltrated four top leaves of each plant. The fourth leaf gave consistently lower expression levels and was not included in the analysis. The three top leaves that were included did not differ significantly from each other (Additional file 2: Figure S4).

## Discussion

Syringe agroinfiltration has been increasingly used as a fast, reliable and low cost method for transient gene expression. The method works particularly well in *N. benthamiana*, but for quantitative assays suffers from a degree of variation. In order to optimize the resources spent for conducting agroinfiltration experiments, we investigated the source of variation using a hierarchical (nested) design in our experiments. A hierarchical design is a special case of a factorial design where the factors do not interact. Instead, errors (variance) is propagated from one hierarchical level up to the next in a simple manner that allows easy calculation of the variance contribution by each nested level. Hierarchical designs are typically used for resource optimisation [31], in biology for example for guiding optimal expenditure for replication in quantitative PCR [32].

We conducted two experiments where activity of an intron containing reporter gene encoding firefly luciferase was used to monitor transient gene expression 2 days after infiltration of *N. benthamiana* leaves with agrobacterium carrying the reporter in its T-DNA. Both experiments showed that the main variation comes from unequal distribution of the reporter activity within an infiltrated leaf. This was somewhat unexpected, and we could not address the variation to uneven spread of agrobacteria in infiltration, variation in the sampling procedure itself or to positional effects of the sampling along the leaf axis. However, in agroinfiltration many errors add up to this particular hierarchical level and may explain together part of the high variation.

A more expected variation, but second to the within leaf variation, was due to the individual leaves infiltrated. We saw usually little variation between plants within a single experiment, although in the first experiment we observed in one case a major difference between the two plants used for infiltration. The second experiment addressed also replication of the infiltration setup (experimental replicates), including a different batch of agrobacterium suspension and different history of the set of plants growing on a shared tray. Variation between the experimental replicates was negligible. Finally, for technical replication of the luciferase assay, we found that using a submicroliter sample of leaf extract caused variation that could be easily avoided by increasing the sample volume.

In the first experiment we used different promoters to drive the luciferase reporter. The promoter choice naturally introduced a large variation in reporter activity, but was included in order to assay for inducibility of the XVE/LexA system and to compare it to the commonly used constitutive 35S promoter. We could measure an eightfold induction by estradiol of the XVE/LexA transcription factor/promoter cassette and the induced levels were about the same or slightly higher than the constitutive levels achieved with the 35S promoter. Zuo and coworkers [27] tested XVE/LexA in stably transgenic Arabidopsis plants with GFP as reporter. Without estradiol induction, GFP mRNA was below the level of detection. Induced with saturated estradiol concentration (5 µM), the induced promoter activity was four times higher than 35S. The G10-90 promoter, in our hands, was much less active than the 35S promoter. Using stably transformed *N. tabacum* and assay for β-glucuronidase enzyme activity encoded by the reporter gene *uidA*, Ishige and coworkers [29] concluded that G10-90 is much stronger than 35S promoter (assayed in cotyledons, roots and seeds).

## Conclusions

We have teased apart the variation in transient agrobacterium infiltration experiments and can come up with recommendations for setting up similar experiments. Most of the variation comes from uneven expression of the reporter gene within a leaf. Therefore, several sampling spots should be combined for the assay. Technical replication of the reporter enzyme assay is not important, if one takes care that pipetting errors are controlled by avoiding submicroliter volumes. The physiological state of the test plant can cause variation. Growth of plants should be standardized and individuals with extreme characteristics should be discarded. In order to monitor the plant parameter, several individuals should be used. In summary, infiltrate more plants but less leaves, sample more positions on the leaf but run only few technical replicates.

## Methods

### Plant material

*Nicotiana benthamiana* plants were grown under fluorescent light at 24 °C in peat: vermiculite (1:1). Day length was 16 h and the relative humidity 65 %. Plants were watered twice a week with commercial fertilizer (Substral, Thompson Siegel, Germany) and used for infiltration at age of 6 weeks when they typically carried nine leaves.

### Construction of plasmids

In order to avoid measuring luciferase activity generated by agrobacterium cells, we used a firefly luciferase cDNA that contains an intron in the coding sequence [33]. The binary plasmid pLKB10, a kind gift from George Allen, contains this reporter under the 35S promoter. In order to generate expression constructs for the first experiment, we amplified the *LUC* gene from pLKB10 using first primers 5′-AAAAAGCAGGCTCCATGGAAGACGCCAAAAAC and 5′-AGAAAGCTGGGTGTTACAATTTGGACTTTC, followed by *att*B adapter primers, as described in the manual for Gateway cloning (Invitrogen). The fragment was inserted to pDONR221 (Invitrogen) using the Gateway BP Clonase enzyme (Invitrogen) to form plasmid pEnLUC.

For generation of the estradiol inducible reporter construct and the G10-90-*LUC* reporter, multisite Gateway cloning was used. The following plasmids were kind gifts from Ari Pekka Mähonen: pEnNosT2-R2R3 containing a nopaline synthase gene polyadenylation site flanked by *att*R2 and *att*L3 sites, pEnPG1090-L4R1 containing the G10-90 promoter flanked by *att*L4 and *att*R1 sites, pEnPG1090XVE-L4R1 containing a G10-90-*XVE* construct, expressing the chimeric estrogen inducible transcription factor XVE [27], followed by the LexA promoter, the cassette flanked by *att*L4 and *att*R1 sites, and pCAMkan-R4R3, which is a pCAM1300 [34] derived Gateway destination vector where *att*R4 and *att*R3 sites flank the *ccdB cam* cassette.

In order to construct the estradiol induced luciferase reporter plasmid pExpXVE-LUC, pEnLUC, pEnNosT2-R2R3, pEnPG1090XVE-L4R1 and pCAMkan-R4R3 were used as substrates in a multisite Gateway reaction catalysed by Gateway LR Clonase. To make pExpG1090-LUC that carries G10-90-*LUC* in its T-DNA, pEnLUC, pEnNosT2-R2R3, pEnPG1090-L4R1 and pCAMkan-R4R3 were similarly combined.

The luciferase reporter was also recombined from pEnLUC to the destination vector pK7WG2D [35] using Gateway LR Clonase. The resulting plasmid pExp35S-LUC, used in the first experiment, is functionally equivalent to pLKB10 that was used in the second experiment. All resulting expression vectors were transformed into the *Agrobacterium tumefaciens* strain C58C1(pGV2260) [36] using electroporation.

### Preparation of Agrobacterium suspension

In addition to the luciferase containing *Agrobacterium tumefaciens* strains described above, we used in the second experiment also C58C1(pGV2260, pBin61-p19) that provides suppression for gene silencing [15]. The purpose was to dilute the luciferase expressing strain so that the luminometer readings would not overflow, suppression of silencing is not needed when the reporter is assayed after only 2 days of expression. Agrobacterium strains were streaked on solid Luria Broth (LB) supplemented with antibiotics (rifampicin, carbenicillin and kanamycin or spectinomycin, all at 100 µg/ml) and grown at 28 °C for 3 days to single colonies. Colonies were inoculated into 5 ml LB with 20 μM acetosyringone and 10 mM 2-(*N*-morpholino) ethane sulfonic acid (MES, pH6.0) without antibiotics, and grown for overnight with vigorous shaking at 28 °C. Cells were collected by centrifugation at 3200×*g* for 10 min at room temperature and resuspended in 2 ml Mg-MES buffer (200 µM acetosyringone, 10 mM MgCl_2_, 10 mM MES, pH 6.0). 200 µl of bacterial suspensions were diluted to 3 ml of Mg-MES buffer and adjusted to a final density of OD_600_ = 0.5. The cell suspensions were kept for 3 h at room temperature before infiltration into tobacco leaves.

### Agroinfiltration of tobacco leaves

Three top leaves of 6 week old *N. benthamiana* plants were used for infiltration, excluding the youngest leaf that was difficult to infiltrate. Agrobacterium suspension was infiltrated into the whole leaf area from a small cut in the lower epidermis, using a 1 ml plastic syringe without a needle. After agroinfiltration, the plants were kept in the growth room for 2 days before harvest. For estradiol induction, the plants were watered with 10 µM 17-β-estradiol (Sigma-Aldrich) 3 days prior to infiltration and subsequently until sampling.

All transgenic material was handled according to the Finnish GMO legislation. The laboratories where this work was conducted has a permanent permission for this type of experiments (Diary number 004/S/2002).

### Determination of luciferase activity

Leaves were sampled from four or five different positions by using a cork bore as a punch. The punched leaf disks were 5.5 mm in diameter and weighed approximately 2.2 mg. Soluble proteins were extracted from the leaf disks using 100 µl of modified lux buffer (50 mM Na-phosphate pH 7.0, 4 % soluble polyvinylpyrrolidone Mw 360,000, 2 mM EDTA, 20 mM DTT) [37], homogenised with a small pestle on ice and centrifuged for 10 min at 4 °C in a microcentrifuge. In the first experiment, luciferase activity was measured in the samples at 24 °C by pipetting 0.5 µl of the supernatant into 50 µl of enzyme substrate (Luciferase 1000 Assay System, #E4550, Promega), fast vortexing and counting photons for 1 s in the luminometer (Luminoskan TL plus, generation II, Thermo Labsystems, Finland). In the second experiment, 10 µl of the supernatant was pipetted into 80 µl of enzyme substrate and photons were counted for 5 s.

### Statistics analysis

Our infiltration experiments are hierarchical (nested) designs that allow calculation of the amount of variance generated at different hierarchical levels of infiltration, sampling or measurement of the luciferase activity. The statistical (linear effects) model used to analyse the nested designs is

$${\mathbf{y}}_{{{\mathbf{ijklm}}}} = {\varvec{\upmu}} + {\mathbf{A}}_{{\mathbf{i}}} + {\mathbf{B}}_{{{\mathbf{ij}}}} + {\mathbf{C}}_{{{\mathbf{ijk}}}} + {\mathbf{D}}_{{{\mathbf{ijkl}}}} + {\mathbf{E}}_{{{\mathbf{ijklm}}}}$$

where **µ** represents the mean of all measurements, **A** the top hierarchical level (promoter in the first experiment and repetition of the infiltration subexperiment in the second experiment), **B** the second hierarchical level (plant treated), **C** the third (leaf infiltrated), **D** the fourth (sample punched) and **E** the residual error, estimated by running technical replicates of the luciferase assay. Calculation of the variance components is explained by Quinn and Keough [38] and shown in Additional file 1: Tables S1, S2.

## Additional files

10.1186/s13007-015-0091-5 Measurements of luciferase activity from the first experiment and calculation of variance components. **Table S2.** Measurements of luciferase activity from the second experiment and calculation of variance components.
10.1186/s13007-015-0091-5 Spread of agrobacterium in leaf infiltration. **Figure S2.** Distribution of luciferase activity within leaves. **Figure S3.** Variation in protein extraction. **Figure S4.** Efficiency of transient expression in different leaves.

## Abbreviations

LB: Luria broth
LexA: an *Escherichia coli* repressor protein
*LUC*: *Photinus pyralis* gene encoding luciferase
MES: 2-(*N*-morpholino) ethane sulfonic acid
T-DNA: transferred DNA of an *Agrobacterium tumefaciens* Ti-plasmid
*uidA*: *Escherichia coli* gene encoding β-glucruonidase
XVE: a chimeric protein consisting of the DNA binding domain of LexA, the transactivating domain VP16 and the regulatory domain of human estrogen receptor
