# Supplementary material for: Within leaf variation is the largest source of variation in agroinfiltration of Nicotiana benthamiana
Source: Plant Methods. 2015 Oct 14;11:47. doi: 10.1186/s13007-015-0091-5 (PMC4607171; doi:10.1186/s13007-015-0091-5)
Supplement: Supplementary file 2 — 10.1186/s13007-015-0091-5 Spread of agrobacterium in leaf infiltration. Figure S2. Distribution of luciferase activity within leaves. Figure S3. Variation in protein extraction. Figure S4. Efficiency of transient expression in different leaves. [file 13007_2015_91_MOESM2_ESM.pdf]

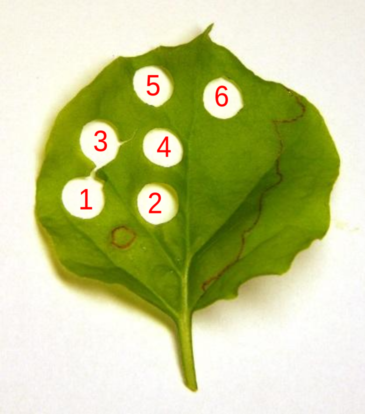

Disk	Bacterial count	
1	400 000	
2	309 000	
3	400 000	
4	409 000	
5	455 000	
6	382 000	
	392 500	Average
	47 694	Std
	12 %	CV

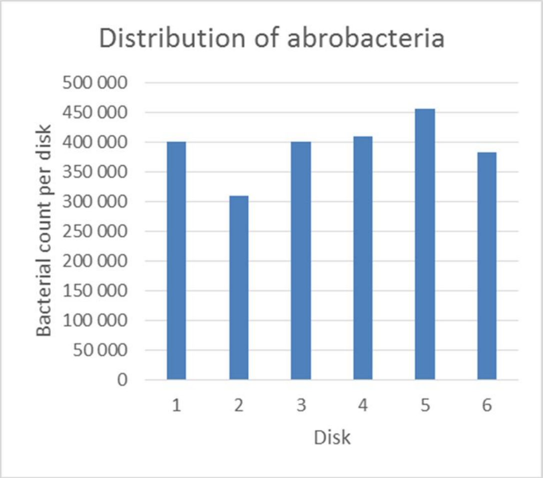

Figure S1. Spread of agrobacterium in leaf infiltration. *Nicotiana benthamiana* leaf was syringe infiltrated with agrobacterium suspension at the position marked with the red circle. Infiltration medium spread to nearly the whole leaf, marked with red ink. After infiltration, six leaf disks were sampled, bacteria were released by homogenization and their count was determined by plating serial dilutions. Agrobacterium spreads evenly in the infiltrated area. Std, standard deviation; CV, coefficiency of variation (Std/Average).

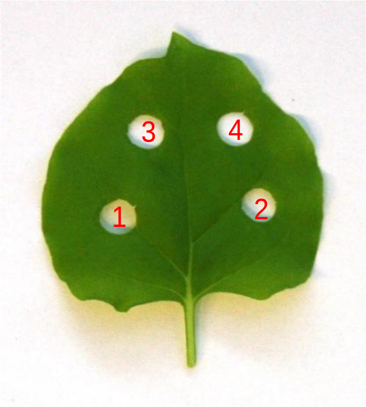

Disk	Average	Std	CV
1	183184	72318	39 %
2	176923	68993	39 %
3	204537	56979	28 %
4	202814	58750	29 %

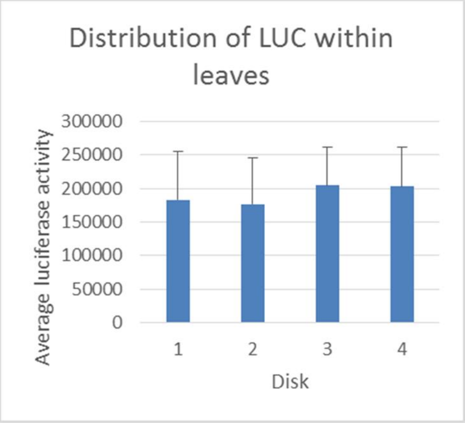

Figure S2. Distribution of luciferase activity within leaves. *Nicotiana benthamiana* leaves were syringe infiltrated with agrobacterium carrying T-DNA with a 35S-*LUC* construct and sampled according to the scheme shown on the left. Average and standard deviation (Std) of the luciferase activity at each sample position, throughout experiment 2, is shown in the table and in the graph. No significant variation between sample points was observed (*P*=0.061). CV of the four disk averages is 7.2% of the grand average. CV, coefficiency of variation (Std/Average).

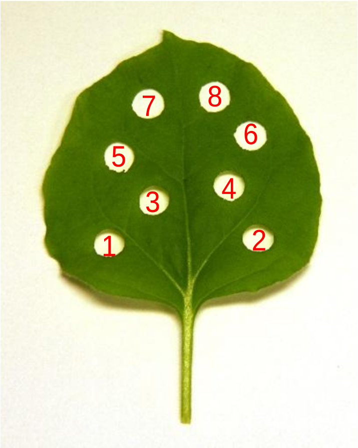

Disk	Protein (µg)	
1	13.7	
2	14.6	
3	14.4	
4	13.7	
5	13.1	
6	14.0	
7	13.8	
8	12.2	
	13.7	Average
	0.75	Std
	5.5 %	CV

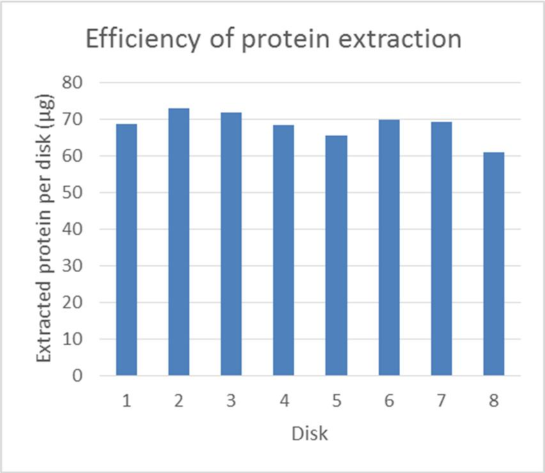

Figure S3. Variation in protein extraction. *Nicotiana benthamiana* leaf was sampled and soluble proteins were extracted as described in the materials and methods for luciferase assay. Soluble protein concentration was measured from cleared homogenates with BSA as standard using the dye binding assay (Biorad). Protein yield showed 5.5 % variation between samples. Std, standard deviation; CV, coefficiency of variation (Std/Average).

Although none of the tested sources contributed a major fraction of the within leaf variance, together they contribute up to 15 %, taken that they are independent of each other ($\sqrt{0.12^2 + 0.055^2 + 0.072^2}$).

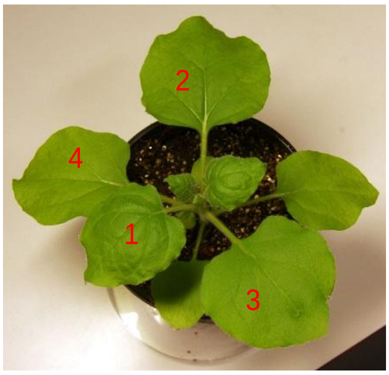

Leaf	Average	Std	CV
1	188085	47577	25 %
2	218945	66536	30 %
3	201412	63150	31 %
4	123435	50309	41 %

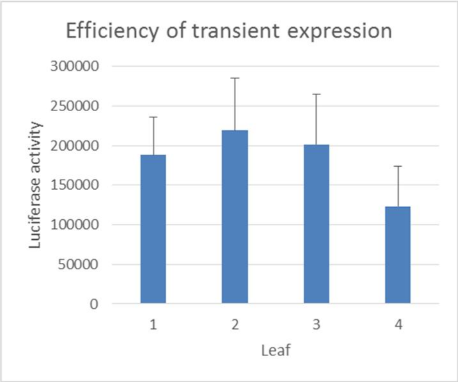

Figure S4. Efficiency of transient expression in different leaves. Four top leaves of *Nicotiana benthamiana*, according to the scheme shown on the left, were syringe infiltrated with agrobacterium carrying T-DNA with a 35S-*LUC* construct and sampled as described in materials and methods. For each leaf position (1-4), luciferase activities were averaged. The three top leaves did not differ significantly for their luciferase activity (*P*=0.554) and were included in the hierarchical variance analysis. Std, standard deviation; CV, coefficiency of variation (Std/Average).
